# Functional, cognitive and psychological outcomes, and recurrent vascular events in Pakistani stroke survivors: a cross sectional study

**DOI:** 10.1186/1756-0500-5-89

**Published:** 2012-02-09

**Authors:** Maria Khan, Bilal Ahmed, Maryam Ahmed, Myda Najeeb, Emmon Raza, Farid Khan, Anoosh Moin, Dania Shujaat, Ahmed Arshad, Ayeesha Kamran Kamal

**Affiliations:** 1 Fellow International Cerebrovascular Translational Clinical Research Program, FCPS Neurology, Stroke Service, Aga Khan University, Karachi, Pakistan; 2 Dept. of Medicine, Aga Khan University, M Sc. Epidemiology and Biostatistics, Karachi, Pakistan; 3 M.B.B.S., Sind Medical College, Karachi, Pakistan; 4 M.B.B.S, Dow University of Health Sciences, Karachi, Pakistan; 5 Medical College, Aga Khan University, Karachi, Pakistan; 6 Ziauddin Medical College, Aga Khan University, Karachi, Pakistan; 7 Aga Khan University, International Cerebrovascular Translational Clinical Research Program and Stroke Services, Karachi, Pakistan; 8 Stroke Service and Clinical Research Programs, Aga Khan University, Karachi, Pakistan

## Abstract

**Background:**

There is little direct data describing the outcomes and recurrent vascular morbidity and mortality of stroke survivors from low and middle income countries like Pakistan. This study describes functional, cognitive and vascular morbidity and mortality of Pakistani stroke survivors discharged from a dedicated stroke center within a nonprofit tertiary care hospital based in a multiethnic city with a population of more than 20 million.

**Methods:**

Patients with stroke, aged > 18 years, discharged alive from a tertiary care centre were contacted via telephone and a cross sectional study was conducted. All the discharges were contacted. Patients or their legal surrogate were interviewed regarding functional, cognitive and psychological outcomes and recurrent vascular events using standardized, pretested and translated scales. A verbal autopsy was carried out for patients who had died after discharge. Stroke subtype and risk factors data was collected from the medical records. Subdural hemorrhages, traumatic ICH, subarachnoid hemorrhage, iatrogenic stroke within hospital and all other diagnoses that presented like stroke but were subsequently found not to have stroke were also excluded. Composites were created for functional outcome variable and depression. Data were analyzed using logistic regression.

**Results:**

309 subjects were interviewed at a median of 5.5 months post discharge. 12.3% of the patients had died, mostly from recurrent vascular events or stroke complications. Poor functional outcome defined as Modified Rankin Score (mRS) of > 2 and a Barthel Index (BI) score of < 90 was seen in 51%. Older age (Adj-OR-2.1, *p *= 0.01), moderate to severe dementia (Adj-OR-19.1, *p *< 0.001), Diabetes (Adj-OR-2.1, *p *= 0.02) and multiple post stroke complications (Adj-OR-3.6, *p *= 0.02) were independent predictors of poor functional outcome. Cognitive outcomes were poor in 42% and predictors of moderate to severe dementia were depression (Adj-OR-6.86, *p *< 0.001), multiple post stroke complications (Adj-OR-4.58, *p *= 0.01), presence of bed sores (Adj-OR-17.13, *p *= 0.01) and history of atrial fibrillation (Adj-OR-5.12, *p *< 0.001).

**Conclusions:**

Pakistani stroke survivors have poor outcomes in the community, mostly from preventable complications. Despite advanced disability, the principal caretakers were family rarely supported by health care personnel, highlighting the need to develop robust home care support for caregivers in these challenging resource poor settings.

## Background

Non communicable diseases including stroke are the leading killers in low and middle income countries like Pakistan [1]. A cross-sectional survey from a multiethnic transitional Pakistani community showed that almost a quarter of the respondents had suffered a cerebrovascular event (either a stroke or a Transient Ischemic Attack [TIA]) [2]. Thus, there is a need to generate regionally specific data from these regions to formulate effective management strategies for stroke survivors.

There are studies done in developed countries exploring the functional and cognitive outcomes of stroke [3-5]. Data from Pakistan is restricted to a few hospital based studies that have reported mortality and acute complications [6-8], but nothing is known of the post hospital outcomes of stroke survivors.

There are reasons to suspect that outcomes from stroke in developing countries like Pakistan may be sufficiently different from the developed world to merit investigation. Stroke etiology is different--intracranial disease being more common [9-12], intracranial hemorrhage (ICH) constitutes a higher proportion of strokes; patients are younger and ethnically distinct. A recent study has highlighted this regional difference in stroke outcomes and mortality reported in various stroke trials [13].

Therefore, the primary objective of this study was to report the functional, cognitive and psychological outcomes of stroke survivors after discharge. Secondary objective was to assess the frequency of recurrent vascular events in this population.

## Methods

### Study design and setting

This is a cross-sectional study. Patients were identified from the Aga Khan University Hospital (AKUH) Karachi, Pakistan. This is a 650-bed, internationally accredited tertiary care hospital that caters to the needs of a large multi-ethnic urban population. The hospital has a dedicated stroke unit run by trained nursing staff and neurologists that deals with 600 plus patients annually.

### Case ascertainment/enrollment strategies

Men and women aged ≥ 18 years, with acute stroke during the study period (January, 2010 to December, 2010) were eligible. All discharges from the stroke neurology service were identified from the medical record section using ICD code 430-438 and the relevant stroke pathways.

Acute stroke was defined by the WHO definition as "rapidly developing clinical signs of focal (at times global) disturbance of cerebral function, lasting more than 24 h or leading to death with no apparent cause other than that of vascular origin". The diagnosis was supported by either a Computed Tomography scan or Magnetic Resonance Imaging.

All the discharges were contacted for interview. Those with whom a telephonic contact could be established within 1-12 months of their index stroke and who gave consent to a verbal interview were enrolled in the study. In those who could not give consent directly, or were aphasic, surrogate consent was sought for interview and primary surrogate caregivers reported on the patient's status.

Those who had died of their index stroke during hospital stay were excluded from this study. Subdural hemorrhages, traumatic ICH, iatrogenic stroke within hospital and all other diagnoses that presented like stroke but were subsequently found not to have stroke were also excluded.

### Data collection instruments

A structured telephonic interview was carried out at 1-12 months post discharge. The questionnaire (Additional file 1) had been translated into Urdu using a translation/back-translation procedure to ensure clarity and consistency. It collected data regarding outcomes and recurrent vascular events since discharge. For patients who had died during this time, a verbal autopsy questionnaire was administered to determine the proximate cause of their death [14]. A trained research officer (physician) established telephonic contact and carried out the interviews. Once the telephonic interviews had been carried out, medical records of these patients were accessed, for information on demographics, stroke subtype and risk factors (Additional file 1).

The following scales were used for assessing the outcomes. For functional Outcome Modified Rankin Score (mRS) and Barthel Index (BI) was used. For depression Beck's Depression Inventory (BDI) with direct questioning was used (for surrogate responders), and for dementia we used the Blessed Dementia Scale (BDS). Screening for recurrent stroke was done using a set of questions based on the Stroke Symptom Questionnaire. Those who had been labeled by a physician as having a recurrent stroke or myocardial infarction (MI) were also included amongst those with recurrent events. Details of our data collection instruments are contained in Additional file 1 along with references of the instruments used.

The protocol was approved by the Ethical Review Committee of the Aga Khan University Hospital **(ERC #: 1541-Neu-ERC-2010)**. Verbal informed consent was taken from all respondents and or their legal surrogate respondent prior to interview since this was a telephonic interview. Written consent could not be taken since these participants were identified via medical record discharges and contacted via phone. The interview contents/form/script were reviewed and approved by the committee and thus verbal consent was approved.

### Data analysis

Reported stroke prevalence and complications of stroke has been ascertained through the literature and found to be 21% [2]. We used the figure of 0.21 for prevalence of exposure, along with 80% power, 0.05 significance level, 5% bond on error, and 20% adjustment for non-response rate give the sample size of 309.

Analysis was carried out using the Statistical Package for Social Sciences (SPSS), version 11.5. Initially descriptive statistics and frequencies were generated. Later data were analyzed using logistic regression. In inferential statistics, continuous variables were checked for their linearity, by doing quartile analysis. Dummy variables were created for variables with more than two categories and the reference group for each variable was defined as the category with the minimal risk for functional, cognitive and psychological outcomes associated with stroke, using previous studies.

Composites were created for functional outcome variable and depression. Poor functional outcome was defined as a composite of mRS > 2 and BI ≤ 90. Depression was labeled if Beck's score was > 10 for those patients who had provided information themselves. For those who had surrogate responders, if at least three of the following four symptoms were reported to be present in the patient, he or she was labeled depressed--anger, flat affect, crying spells, and sleeplessness/low appetite. Score of 6-12 was taken as moderate and > 12 as severe dementia on BDS.

A composite variable of recurrent stroke was generated with physician confirming stroke as outcome or if the patient reported permanent neurological deficits (hemiparesis, hemianopsia, monocular blindness, facial deviation or dysphagia, dysarthria). Physician report of angina or MI was also added to the recurrent stroke variable to form a composite of recurrent vascular event.

Multicolinearity was checked among all the independent variables. A univariate logistic regression analysis was conducted to assess the (crude) association of each independent factor with all three outcomes (Additional file 1). Biological significance and a value of p value 0.25 were considered as criteria for a variable to be significant in univariate analysis. Biologically plausible interactions among variables and confounding were also checked. Multivariable logistic regression analysis was done and adjusted odds ratios (ORs) were calculated (Additional file 1).

## Results

Data was collected from patients discharged from AKUH stroke service between January and December, 2010. During this period 650 patients were admitted with acute cerebrovascular event. Subdural hemorrhages (n = 21) and in-hospital expiries (n = 45) were excluded. All the other stroke patients/surrogate caregivers were then contacted for telephonic interviews over a period of 3 months from November 1^st ^to January 31^st^. Three hundred and nine patients/their surrogates consented and were included in the study. Median time from onset of stroke to outcome assessment was 5.5 months.

Demographic and stroke characteristics are described in Table 1. Of the 309 patients included, 62.1% were women. Mean age was 61.75 years (IQR 21-90). Of the 271 patients alive at the time of follow-up, all except one were being taken care of at home, and mostly (56.3%) by family members. None of the patients were in institutions or rehabilitation centers despite the disability status.

**Table 1:** Baseline characteristics of study subjects

Characteristic	Number	(%)
*Gender*		
Male	117	(37.9)
Female	192	(62.1)

*Age*		
≤ 60	150	(48.5)
> 60	159	(51.5)

*Type of stroke (n = 309)*		
Ischemic	241	(78)
Hemorrhagic	68	(22)

*TOAST (n = 241)*		
Large Artery	109	(35.3)
Cardioembolic	41	(13.3)
Small artery Lacune	55	(17.8)
Others	36	(11.6)

*Risk Factors (n = 309)*		
HTN	288	(93.2)
Dyslipidemia	223	(72.2)
DM	178	(57.6)
Obesity (BMI > 25)	124	(40.1)
CAD	110	(35.6)
Smoking/chewed tobacco	60	(19.4)
Intracranial atherosclerosis	85	(28.4)
Atrial fibrillation	37	(12)
Extracranial Carotid stenosis	21	(6.8)
Depression/Anxiety	18	(5.8)
Prior TIA or stroke	52	(16.8)
EF < 30%	19	(6.1)

*Dead (n = 309)*	38	(12.3)

*Care being given (n = 271)*		
At home	270	(99.6)
Readmitted to hospital	1	(0.4)

*Care provision arrangements (n = 270)*		
Independent	91	(33.7)
Family member	152	(56.3)
Professional Nursing	24	(8.9)
Non professional help	3	(1.1)

Majority of the strokes were ischemic (78%). Of these, large artery atherosclerotic disease was the predominant etiologic subtype (35.3%). Hypertension was the commonest risk factor (93.2%), followed by dyslipidemia (72.2%), diabetes (57.6%) and obesity (40.1%).

Forty five (6.9%) of the 650 total patients had died during the hospital admission period. Of the remaining, 309 (51%) could be contacted. Thirty eight of these (12.3%) had died at the time of outcome assessment. Of these hemorrhagic strokes accounted for 26% of index strokes, while the rest were ischemic (12 were partial anterior circulation strokes, 10 were posterior circulation and 6 were lacunar strokes). Verbal autopsy revealed the cause of death as vascular (stroke, MI or both) in 78% of these patients, with recurrent stroke being responsible for 65% of these mortalities. In almost one third of the patients (31.5%) preventable complications, mostly infections related to stroke were important contributors to mortality and in four patients these were the main cause of death as presented in Table 2.

**Table 2:** Stroke outcomes

Outcome variable	Number	(%)
*Mortality*	38	(12.3)
Vascular death	30	(78.9)
Recurrent CVA	16	(53.3)
Myocardial infarction	5	(16.7)
Both	9	(30.0)
Stroke related complications	12	(31.5)
Infections	8	(21)
Seizures	3	(7.9)
Others	1	(2.6)

*Post stroke Complications*	176	(64.9)
Pain	126	(46.5)
Constipation	91	(33.6)
UTI	43	(15.9)
Pneumonia	14	(5.2)
Other infections	11	(4.1)
Seizures	20	(7.4)
Bedsores	17	(6.3)
DVT/PE	4	(1.5)

*Functional Outcomes*		
Modified Rankin Score (n = 271)		
mRS ≤ 2	174	(64.2)
mRS > 2	97	(35.8)
Barthel Index (n = 271)		
Barthel Index 0-30	43	(15.9)
Barthel Index35-60	31	(11.4)
Barthel Index 65-90	41	(15.1)
Barthel Index 95-100	156	(57.6)

*Cognitive Outcomes (n = 271)*		
None BD = 0	31	(11.4)
Mild BD 1-5	126	(46.5)
Moderate BD 5.5-12	77	(28.4)
Severe BD > 12	37	(13.7)

*Psychological Outcomes n = 271)*		
Depressed	57	(21)

*Recurrent Vascular Events (n = 271)*	66	(24.4)
Recurrent Stroke	62	(22.9)
MI/angina	9	(3.3)

When we look at long term mortality according to stroke subtype we find that 9/38 (23%) of ICH patients and 29/38 (76%) ischemic stroke patients died. Of ICH patients, which are hypertensive basal ganglia ICH, 9/64 (14%) died, and of ischemic stroke 29/245(11.8%) died. When we look at how ischemic stroke subtype by TOAST criteria affects mortality in this group, mortality was as follows: Large Artery Atherosclerosis 11/98 (11.2%), Lacunes 4/51 (7.8%), Cardioembolic stroke 9/32 (28%), unspecified 4/30 (13%), there were no mortalities in the 'other specified' group.

Of the 271 patients alive at the time of interviews, 64.9% reported at least one complication since discharge. Pain was the commonest complication present in 126 (46.5%) patients followed by constipation (33.6%) and urinary tract infection (15.9%).

Of those alive, 34.8% had moderate to severe disability defined as mRS > 2. BI indicated that 57.6% were independent and 15.9% were severely disabled (BI ≤ 30).

**Figure 1:**
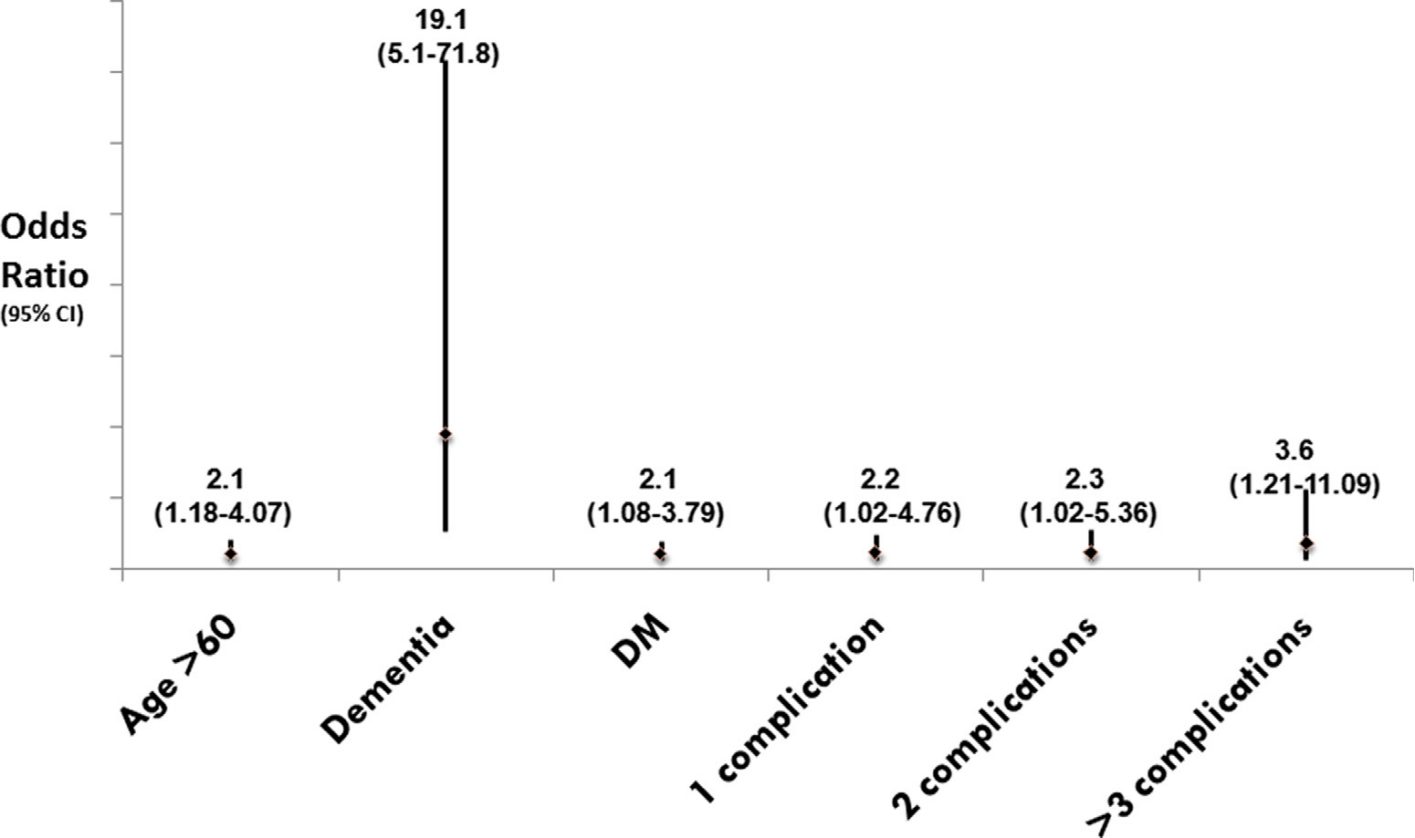
**Poor Functional Outcome: Age, Dementia, Diabetes and post stroke complications were associated with poor functional outcomes**.

When a composite of mRS > 2 and BI ≤ 90 was taken, 51.1% of the patients had poor functional outcomes. Univariate analysis for predictors of poor outcome can be reviewed in our on-line supplement (Additional file 1). The following factors were independently associated with the odds of a poor outcome, older age (OR = 2.1, CI-1.18-4.07), Diabetes (2.1, 1.08-3.79), dementia (19.1, 5.1-71.8), post discharge complications and their increasing multiplicity (3.6, 1.21-11.09) (Figure 1).

**Figure 2:**
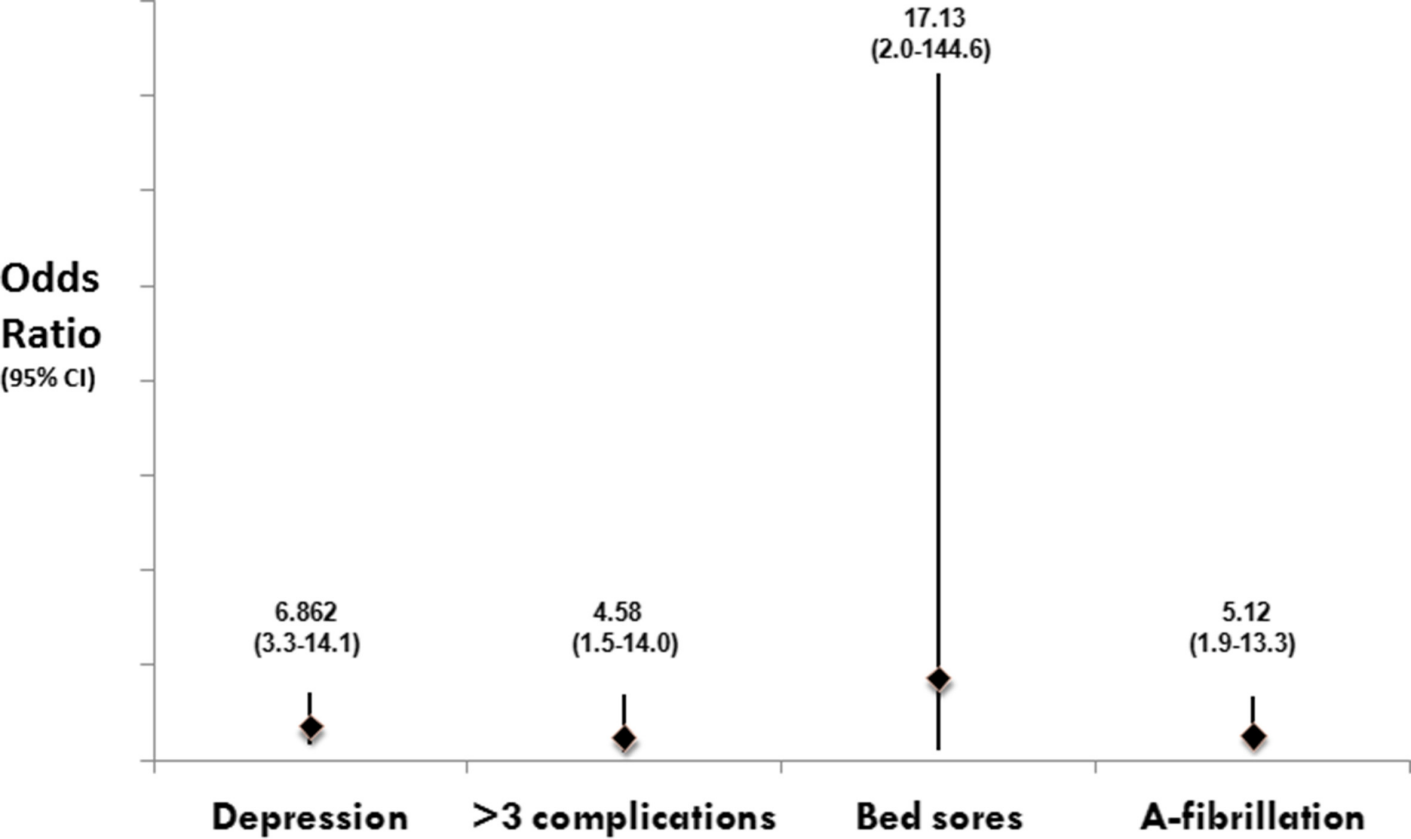
**Moderate to Severe Dementia: Depression, post stroke complications, bedsores and atrial fibrillation uA-Fibrillation) was associated with moderate to severe dementia**.

Moderate to severe dementia defined as BDS score of more than 5 was found in 114 (42.1%) of the 271 patients alive at the time of follow-up. Moderate to severe dementia was more likely in patients who were depressed (OR = 6.86, CI = 3.3-14.1), had 3 or more post stroke complications (4.58, 1.5-14), had bedsores (17.137, 2.0-144.6), and had atrial fibrillation (5.12, 1.9-13.3) (Figure 2).

Overall, 57 of the 271 surviving patients (21%) were depressed. When depression was evaluated, people with moderate to severe dementia were 16.6 times more likely to be depressed.

Sixty six (24.4%) of the 271 patients alive at the time of follow-up reported recurrent vascular events (stroke, MI or both). Stroke was the most common recurrent event (62/271, 22.9%), making up 93% of recurrent events. Of the recurrent strokes, 37 had been confirmed by physicians.

## Discussion

We found that within a median of 5.5 months after discharge at least one third of stroke survivors in Pakistan had either died due to vascular causes or suffered a recurrent vascular event usually a stroke. Despite sustained disability the patients were homebound and cared for by family members with infrequent health personnel support. Poor functional outcome was associated with patient characteristics like old age, Diabetes, CAD, atrial fibrillation, stroke subtype mainly cardioembolic and medically preventable events like post stroke complications and their increasing multiplicity. About half the survivors had moderate to severe dementia and a quarter were depressed.

Our in-hospital mortality is comparable to Western figures with around 6% patients dying during index hospitalization for stroke [15]. We report a 12% all cause mortality following hospital discharge at a median of 5 months. A large inter-study variability exists in international literature (Additional file 1) with 30 day mortality ranging between 5% and 25% [16-21] and one year mortality of 17-24% [16-20]. Regional data reports a much higher 28 day case fatality of 29.8% and 41% [22,23]. When we look at ischemic stroke subtypes and mortality, we find that cardioembolic strokes result in the greatest mortalities in this study, which is comparable to what is reported in the literature. Often the outcome is better in lacunar strokes than non-lacunar strokes [24-28]. However, while making these inferences and the ones that follow, caution must be applied as our study is a cross sectional one and a prospective cohort design would better reflect outcomes.

Around one half of our patients had poor functional outcomes based on mRS and BI. Studies from neighboring India report a 38.5% moderate to severe disability in their stroke survivors based on mRS [22]. Spain reports 37.7% functional dependence based on mRS [3]. Compared to these figures, our functional outcomes are worse. Predictors of poor functional outcomes in our study were older age, dementia and presence of post stroke complications which are consistent with what has been reported in other studies [29-32].

Pooled data from 14 studies on acute stroke reports a 21.7% prevalence of depression post stroke [33] which is comparable to our rate of 21%. Similarly, moderate to severe dementia and its predictors (old age and atrial fibrillation) were comparable to reported rates [34]. Of note was the strong association seen between depression and moderate to severe dementia (OR-16.6) which has also been previously reported [35]. One mechanism suggested for this association is stroke resulting in fronto-subcortical dysfunction that gives rise to depressive symptoms as well as dementia [36].

A quarter of our surviving patients suffered from recurrent vascular events mostly strokes after discharge at a median of 5.5 months post discharge. This figure is much more than what is reported from other studies from around the world with 1 year rates in the range of 5.8-13.3% [17,19,37] (Additional file 1). A potential explanation for this high stroke recurrence rate is the high number of patients with intracranial atherosclerotic disease (ICAD) (35%) in our sample. ICAD is known to have the highest rate of recurrent stroke of around 14% per year [38].

Our study is the first systematic investigation of the state of stroke survivors in a low and middle income country like Pakistan. Its strengths are its comprehensive approach, its coverage and access to all patient and uniformity of acute care. The interviewer was a single trained physician following a tested refined questionnaire, with internationally standardized tools of assessment [39-54].

There are several limitations. First and foremost, we were unable to contact nearly half the patients who were discharged alive. This could have skewed our results in either direction. Secondly, this is a single centre study and the care that these patients received may bias towards better functional outcomes. Thirdly, since this was a cross-sectional study we do not have longitudinal data on outcomes of individual patients. It is known that improvement in functional status continues to happen for 3-6 months after stroke and some of those interviewed earlier may still have been improving at the time of interview. Our sub-analysis however did not show any significant difference in outcomes of patients interviewed at 1-5 months and those interviewed later. Fourthly, although the outcome scales that we used have been validated for telephonic interviews, Urdu translation/cross-cultural factors may have affected results. Also, difficulty in data collection and quality rating were not evaluated. However, after pre-testing the Urdu version, the interviewer made sure that appropriate "trigger words" were used to avoid translational communication errors [55]. Direct observation and examination may have uncovered more cognitive issues and depression than reported. Surrogate responders may have introduced bias in reporting for depression outcomes.

## Conclusions

To conclude, our study has provided valuable insight into what happens to stroke survivors in low and middle income countries once they leave the hospital. Even gains achieved in a dedicated stroke unit are diluted. Physicians and caregivers both need to focus on preventable post stroke complications. In addition, a public health approach to broader preventive measures to avoid catastrophic disabling strokes will also be a viable way forward.

Solutions for the current resource poor situation include care giver training for both rehabilitation and skills to recognize cognitive and psychological complications before the patient goes home. Future trials that assess the impact of caregiver education and support, home based rehabilitation and community based reintegration of Pakistani stroke survivors are likely to have broader relevance in this region.

## Competing interests

The authors declare that they have no competing interests.

## Authors' contributions

MK, Conducted the study, developed the protocol, wrote the manuscript and secured the funding as above. BA, Conceived and performed all statistical analysis and provided epidemiologic feedback on the manuscript. MA, Participated with interviews and data collection. MN, Participated with data collection and entry processes. ER, Participated with data collection and entry processes. FK, Participated with data collection and entry processes. AM, Participated with data collection and entry processes. DS, Participated with data collection and entry processes. AA, Participated with data collection and entry processes. AKK, Conceived the idea, provided overview with study protocol, questionnaire design, analysis and manuscript writing. All authors read and approved the final manuscript.

## Supplementary Material

Additional file 1 **Annexure 1, 2, 3, 4, 5 and 6**. Click here for file
