# Supplementary material for: Functional, cognitive and psychological outcomes, and recurrent vascular events in Pakistani stroke survivors: a cross sectional study
Source: BMC Res Notes. 2012 Feb 9;5:89. doi: 10.1186/1756-0500-5-89 (PMC3296616; doi:10.1186/1756-0500-5-89)
Supplement: Additional file 1 — Annexure 1, 2, 3, 4, 5 and 6. [file 1756-0500-5-89-S1.DOC]

**ONLINE SUPPLEMENT**

**Annexure 1:**

**Questionnaire for Telephonic Assessment:**

**OUTCOME ASSESSMENT AFTER STROKE**

Name: _________________ MR#: _______________ Gender: _____ Age: _____

Profession_________________ Address ____________________

Date of Stroke: ___________ Date of Follow up: ______________

Stroke Subtype: ICH: Ischemic stroke:

__________________________________________________________________________________

1. If Respondent is not the patient:_________ (mention relationship)
2. Is the patient alive? Y⁭ N⁭ If no then go to the Verbal autopsy form
3. Baseline mRS? ___________
4. Care being given?
5. At home
   - - - 1. no support needed
         2. with family members
         3. with nursing support
6. At a nursing home
7. Re-admitted to a hospital
8. Back to work
9. What were the complications seen after the discharge?
10. Seizures Y⁭ N⁭
11. Infections: a) UTI Y⁭ N⁭

b) Aspiration Pneumonia Y⁭ N⁭

c) Others Y⁭ N⁭

1. Immobility a) DVT/PE Y⁭ N⁭

b) Bed sores Y⁭ N⁭

c) Constipation Y⁭ N⁭

1. Pain Y⁭ N⁭
2. **Functional Outcome:**
3. **MODIFIED RANKIN SCORE**

**mRS at discharge:___________**

**mRS now: __________**

1. **BARTHEL INDEX SCORE**

1. **Cognitive Outcome:**

1. **PSYCHOLOGICAL OUTCOME**

1. **SCREENING FOR RECURRENT CEREBROVASCULAR EVENTS:**
2. RECURRENT STROKE/TIA

During this follow up period:

1. Were you told by a physician that you have had a new stroke? Y N
2. Did you have a sudden painless loss of vision? Y N
3. If yes, was this transient and for what duration? Y N
4. Did you suddenly lose one half of your vision? Y N
5. If yes, was this transient and for what duration? Y N
6. Did you suddenly develop numbness of one half of your body? Y N
7. If yes, was this transient and for what duration? Y N
8. Did you suddenly develop weakness of one half of your body? Y N
9. If yes, was this transient for what duration? Y N
10. Did you suddenly develop difficulty in walking? Y N
11. If yes, was this transient and for what duration Y N
12. Did you suddenly develop facial deviation? Y N
13. If yes, was this transient and for what duration? Y N
14. Did you suddenly develop new onset difficulty in swallowing or speaking? Y N
15. If yes, was this transient and for what duration? Y N
16. CARDIAC EVENTS

During this follow up period:

1. Were you told by a physician that you had Angina or a Heart Attack? Y⁭ N⁭
2. Did you ever have any pain or discomfort in your chest? Y⁭ N⁭
3. Does walking on a level surface at ordinary pace produce chest pain? Y⁭ N⁭
4. Does walking uphill or at a hurried pace produce chest pain? Y⁭ N⁭
5. **VERBAL AUTOPSY:**

**Annexure 2:**

**Data Collection Form for Medical Chart Review:**

**OUTCOME ASSESSMENT OF PATIENTS-CHART REVIEW**

**MR#: __________________ Baseline mRS: _________**

**Patient Occupation Prior to Stroke**: Retired Working

**Type of stroke**: a) Ischemic b) Hemorrhagic

**Oxfordshire Classification for Ischemic Stroke**

1. TACS c)PACS
2. LACS d)POCS

**TOAST Classification**

1. Large Artery Atherosclerosis
2. Cardioembolic
3. Small Artery Lacunar
4. Other specified
5. Unspecified

**Risk Factors**

1. Hypertension ( >140/90 during chart review or Known or on antihypertensives )
2. Diabetes Mellitus ( Abnormal HBAIC during review, known or on Diabetes drugs)
3. Coronary Artery Disease ( MI , Angina , CABG, CHF , cardiac medications )
4. Dyslipidemia ( Cholesterol > 200 or HDL < 35 or LDL> 100 )
5. Smoking
6. Chewed tobacco
7. Alcohol Use
8. Atrial fibrillation ( Known or discovered during EKG or Holter Monitor for stroke)
9. Carotid stenosis( Known or discovered during the hospital stay )
10. Intracranial Atherosclerosis ( On MRA )
11. Transient Ischemic Attack
12. Depression/Anxiety
13. Obstructive Sleep Apnea
14. Obesity : By weight, waist/ hip or BMI ( from nursing documentation )
15. Previous known stroke clinical
16. Family History of Stroke
17. Hemosiderin deposits on the MRI , Heme sequence , Cerebral microbleeds noted
18. EF<30%
19. Valvular heart disease

**Annexure 3: Tools used in Outcome Assessment**

The following scales were used for assessing the outcomes.

**Functional Outcome:**

***Modified Rankin Scale***: The Modified Rankin Scale remains the most prevalent functional outcome measure in stroke studies. Its main merit lies in its simplicity and telephonic assessment can be done with ease (1-4). It is a six point scale, 0-6 with higher numbers denoting greater functional dependence and 6 denoting death. For the purpose of our analysis mRS of 2 or less was classified as good outcome and 3-6 as poor functional outcome.

***Barthel*** ***Index*:** The Barthel Index (BI) is considered a valid and reliable measure of the degree of independence in daily activities achieved following strokes. It has been shown to have a good correlation between performance-based scoring and interview-based scoring. In addition, the high validity observed in the telephone interview supports its use in longitudinal studies and large surveys where direct performance evaluation is not feasible or too costly (5-8). BI is a 100 point score which measures a person’s functioning in 10 domains of activities of daily living and mobility. Higher scores signify greater ability to live independently. For our analysis, a BI of 95 and above was taken as indicator of good functional outcome.

**Cognitive Outcome:**

***Blessed Dementia Scale****:* Our reason for choosing this scale for cognitive assessment is its reliability, simplicity and ease of being able to assess based on telephonic replies (9-12). BDS is a 28 point score, with scores 0-5 signifying mild cognitive impairment, 6-12 signifying moderate impairment, and >12 signifying severe dementia.

**Depression:**

***Beck’s Depression Inventory:*** The Beck Depression Inventory is a 21-item questionnaire commonly used in research on post stroke depression. It has been validated in a study using other Depression inventories as well (13). A score of 10 or more indicates depression. Our reason for using this particular scale is its reliability, and again the ease with which it can be assessed over the telephone (13-16). For patients who were unable to answer for themselves for any reason, a separate set of questions were asked from the surrogate respondent to assess depression. These questions included anger, flat affect, crying spells and decreased sleep/appetite. The patient was labeled as being depressed if the answer to 3 out of these four questions was in the affirmative.

**Recurrent Vascular Events:**

For screening for recurrent stroke, a set of questions based on the Stroke Symptom Questionnaire were asked from the respondents. Those who had been labeled by a physician as having a recurrent stroke or myocardial infarction were also included amongst those with recurrent events (17).

**Verbal Autopsy:**

For patients who had died since discharge, a sensitive verbal autopsy was carried out to determine the proximate cause of death (18). This verbal autopsy has been validated for vascular deaths.

**References:**

- - - 1. Rankin J. Cerebral vascular accidents in patients over the age of 60. I. General considerations. Scott Med J. 1957;2(4):127-36.
      2. Bonita R, Beaglehole R. Recovery of motor function after stroke. Stroke. 1988;19:1497-500.
      3. Van Swieten JC, Koudstaal PJ, Visser MC, Schouten HJ, van Gijn J. Interobserver agreement for the assessment of handicap in stroke patients. Stroke. 1988;19:604-7.
      4. Quinn TJ, Dawson J, Walters MR, Lees KR. Reliability of the modified Rankin Scale: a systematic review. Stroke. 2009;40:3393-5.
      5. Mahoney FI, Barthel DW. Functional Evaluation: The Barthel Index. Md State Med J. 1965;14:61-5.
      6. Shinar D, Gross CR, Bronstein KS, Licata-Gehr EE, Eden DT, Cabrera AR, et al. Reliability of the activities of daily living scale and its use in telephone interview. Arch Phys Med Rehabil. 1987;68:723-8.
      7. Gresham GE, Phillips TF, Labi ML. ADL status in stroke: relative merits of three standard indexes. Arch Phys Med Rehabil. 1980;61:355-8.
      8. Collin C, Wade DT, Davies S, Horne V. The Barthel ADL Index: a reliability study. Int Disabil Stud. 1988;10(2):61-3.
      9. Blessed G, Tomlinson BE, Roth M. The association between quantitative measures of dementia and of senile change in the cerebral grey matter of elderly subjects. Br J Psychiatry. 1968;114:797-811.
      10. Stern Y, Hesdorffer D, Sano M, Mayeux R. Measurement and prediction of functional capacity in Alzheimer's disease. Neurology. 1990;40:8-14.
      11. Zillmer EA, Fowler PC, Gutnick HN, Becker E. Comparison of two cognitive bedside screening instruments in nursing home residents: a factor analytic study. J Gerontol. 1990;45:69-74.
      12. Madureira S, Guerreiro M, Ferro JM. Dementia and cognitive impairment three months after stroke. Eur J Neurol. 2001;8:621-7.
      13. Aben I, Verhey F, Lousberg R, Lodder J, Honig A. Validity of the beck depression inventory, hospital anxiety and depression scale, SCL-90, and hamilton depression rating scale as screening instruments for depression in stroke patients. Psychosomatics. 2002;43:386-93.
      14. Beck AT, Ward CH, Mendelson M, Mock J, Erbaugh J. An inventory for measuring depression. Arch Gen Psychiatry. 1961;4:561-71.
      15. Richter P, Werner J, Heerlein A, Kraus A, Sauer H. On the validity of the Beck Depression Inventory. A review. Psychopathology. 1998;31:160-8.
      16. Hosking SG, Marsh NV, Friedman PJ. Poststroke depression: prevalence, course, and associated factors. Neuropsychol Rev. 1996;6:107-33.
      17. Berger K, Hense HW, Rothdach A, Weltermann B, Keil U. A single question about prior stroke versus a stroke questionnaire to assess stroke prevalence in populations. Neuroepidemiology. 2000;19:245-57.
      18. Truelsen T, Heuschmann PU, Bonita R, Arjundas G, Dalal P, Damasceno A, et al. Standard method for developing stroke registers in low-income and middle-income countries: experiences from a feasibility study of a stepwise approach to stroke surveillance (STEPS Stroke). Lancet Neurol. 2007;6:134-9

**Annexure 4: Stroke Outcomes in International Studies**

| **Author, year** | **Type of study** | **No. of patients** | **Duration of follow-up** | **Vascular Deaths (%)** | **Recurrent Strokes** | | **MI** |
| --- | --- | --- | --- | --- | --- | --- | --- |
| ***Nagasawa et al 2011^1^*** | Single centre/ Japan | 548 | 17 mo | 7.1 |  |  | |
| ***Fjaertoft, 2011^2^*** | Single centre/ Norway | 306 | 5 yr | 48.4 |  |  | |
| ***Lakshminarayan, 2011^3^*** | Medicare based >65, Minnesotta | 879 | 30 day | 11.1 | 1.8 | 0.6 | |
|  |  |  | 1 year | 24 | 5.8 | 2.1 | |
|  |  |  | 5 year | 49 | 20.7 | 5.7 | |
| ***Saposnik, 2011^4^*** | Registry of Canadian Stroke Network, excluded hemorrhagic strokes | 8223 | 30 day | 12.2 |  |  | |
|  |  |  | 1 year | 22.5 |  |  |  |
| ***Feng, 2010^5^*** | Hospitalized patients, South Carolina | 10,339 | 30 day | 11.4 | 1.8 | 0.3 | |
|  |  |  | 6 mo | 14.8 | 5 | 1 | |
|  |  |  | 1 year | 17.1 | 8 | 2.1 | |
|  |  |  | 4 year | 26.7 | 18.1 | 6.2 | |
| ***Dhamoon, 2006-***  ***2007(Northern Manhattan Stroke Study-first ischemic stroke-NOMAS)^6-7^*** | Community based study, Northern Manhattan | 655 | 30 day | 5.3 | 1.2 | 0.2 | |
|  |  |  | 1 year | 6.5 | 6.6 | 1.5 | |
| ***Azarpazhooh, 2008 (NEMESIS)^8^*** | Community based, Melbourne, Australia | 1316 | 2 year |  | 7.8 |  | |
| ***Hardie, 2004, Perth Community Stroke study-first ever stroke^9^*** | Community based, Perth, Australia | 251 | I yr |  | 11.9 |  | |
|  |  |  | 10 yr |  | 25.5 |  |  |
| ***Tseng, 2009(readmission data)^10^*** | National Health Insurance Database, Taiwan | 468 | 1 yr |  | 13.3 |  | |

**References:**

1. Nagasawa H, Yokota C, Toyoda K, Ito A, Minematsu K. High level of plasma adiponectin in acute stroke patients is associated with stroke mortality. J Neurol Sci. 2011;304:102-6.
2. Fjaertoft H, Rohweder G, Indredavik B. Stroke unit care combined with early supported discharge improves 5-year outcome: a randomized controlled trial. Stroke. 2011;42:1707-11.
3. Lakshminarayan K, Schissel C, Anderson DC, Vazquez G, Jacobs DR, Jr., Ezzeddine M, et al. Five-Year Rehospitalization Outcomes in a Cohort of Patients With Acute Ischemic Stroke: Medicare Linkage Study. Stroke. 2011; 42:1556-1562
4. Saposnik G, Kapral MK, Liu Y, Hall R, O'Donnell M, Raptis S, et al. IScore: a risk score to predict death early after hospitalization for an acute ischemic stroke. Circulation. 2009;123:739-749
5. Feng W, Hendry RM, Adams RJ. Risk of recurrent stroke, myocardial infarction, or death in hospitalized stroke patients. Neurology. 2010;74:588-93.
6. Dhamoon MS, Sciacca RR, Rundek T, Sacco RL, Elkind MS. Recurrent stroke and cardiac risks after first ischemic stroke: the Northern Manhattan Study. Neurology. 2006;66:641-6
7. Dhamoon MS, Tai W, Boden-Albala B, Rundek T, Paik MC, Sacco RL, et al. Risk of myocardial infarction or vascular death after first ischemic stroke: the Northern Manhattan Study. Stroke. 2007;38:1752-8.
8. Azarpazhooh MR, Nicol MB, Donnan GA, Dewey HM, Sturm JW, Macdonell RA, et al. Patterns of stroke recurrence according to subtype of first stroke event: the North East Melbourne Stroke Incidence Study (NEMESIS). Int J Stroke. 2008;3:158-64.
9. Hardie K, Hankey GJ, Jamrozik K, Broadhurst RJ, Anderson C. Ten-year risk of first recurrent stroke and disability after first-ever stroke in the Perth Community Stroke Study. Stroke. 2004;35:731-5.
10. Tseng MC, Lin HJ. Readmission after hospitalization for stroke in Taiwan: results from a national sample. J Neurol Sci. 2009;284:52-5.

**Annexure 5: Univariate Logistic regression Analysis of Functional, Cognitive and Psychological Outcomes in Pakistani Stroke Survivors**

| **UNIVARIATE ANALYSIS** | | | | | | | | | |
| --- | --- | --- | --- | --- | --- | --- | --- | --- | --- |
|  | **Functional** | | | **Dementia** | | | **Depression** | | |
| **Variable** | **Un-Adjusted Odds Ratios** | **95% CI** | **P-Value** | **Un-Adjusted Odds Ratios** | **95% CI** | **P-Value** | **Un-Adjusted Odds Ratios** | **P-Value** | **95% CI** |
| **Age** |  |  |  |  |  |  |  |  |  |
| ≤ 60 | Reference |  |  | Reference |  |  | Reference |  |  |
| > 60 | 2.2 | (1.4-3.6) | 0.00 | 1.68 | (1.03 - 2.06) | 0.03 | 1.01 | 0.95 | (0.5-1.8) |
| **Gender** |  |  |  |  |  |  |  |  |  |
| female | Reference |  |  | Reference |  |  | Reference |  |  |
| male | 0.71 | (0.4-1.1) | 0.14 | 0.88 | (0.53- 1.45) | 0.62 | 0.93 | 0.81 | (0.5-1.6) |
| **Stroke subtype** |  |  |  |  |  |  |  |  |  |
| ischemic | Reference |  |  | Reference |  |  | Reference |  |  |
| ICH | 0.74 | ( 0.4-1.2) | 0.29 | 0.94 | (0.50- 1.75) | 0.85 | 0.92 | 0.83 | (0.4-1.9) |
| **Complications** |  |  |  |  |  |  |  |  |  |
| no | Reference |  |  | Reference |  |  | Reference |  |  |
| yes | 3.8 | ( 2.2-6.9) | 0.00 |  |  |  | 1.3 | 0.35 | (0.7-2.5) |
| **TOAST** |  |  |  |  |  |  |  |  |  |
| small artery | Reference |  |  | Reference |  |  | Reference |  |  |
| large artery | 1.5 | ( 0.7 - 2.9 ) | 0.5 | 1.06 | (0.5 - 2.1) | 0.86 | 0.7 | 0.5 | (0.3-1.8) |
| cardio embolic | 5.7 | (2.2-14.4) | 0.00 | 3.23 | (1.2 - 8.1) | 0.01 | 1.2 | 0.7 | (0.4-3.4) |
| other specified | 1.6 | (0.09-27.2) | 0.6 |  |  |  | 3.6 | 0.3 | (0.2-62.9) |
| unspecified | 2 | (0.8-4.8) | 0.33 | 2.38 | (0.9 - 6.0) | 0.06 | 1.5 | 0.4 | (0.5-4.3) |
| **Complications after discharge** |  |  |  |  |  |  |  |  |  |
| no | Reference |  |  | Reference |  |  | Reference |  |  |
| yes | 3.8 | (2.2-6.6) | 0.00 | 3.8 | (2.1-6.8) | 0.00 | 1.3 | 0.3 | (0.7-2.5) |
| **Complications** |  |  |  |  |  |  |  |  |  |
| Seizures | 1.9 | (0.7-5.0) | 0.14 | 1.8 | (0.7-4.5) | 0.2 | 0.9 | 0.9 | (0.3 - 2.9) |
| UTI | 3.5 | (1.7-7.1) | 0.00 | 3.6 | (1.8-7.2) | 0.00 | 1.3 | 0.4 | (0.6-2.9) |
| Pneumonia | 8.3 | (1.8-38.1) | 0.00 | 3.8 | (1.1-12.4) | 0.02 | 2.1 | 0.1 | (0.7 - 6.7) |
| infections- others | 1.5 | (0.4-5.2) | 0.47 | 1.8 | (0.4-6.9) | 0.3 | 1.4 | 0.6 | (0.3-5.5) |
| thrombosis | 3.9 | (0.4-38.1) | 0.24 | 4.4 | (0.4-42.8) | 0.2 |  |  |  |
| bedsores | 10.7 | (2.4-47) | 0.00 | 24.5 | (3.1-189.0) | 0.00 | 1.1 | 0.8 | (0.3-3.7) |
| constipation | 2.3 | (1.4-3.9) | 0.00 | 1.55 | (0.9-2.6) | 0.09 | 1.2 | 0.5 | (0.6-2.2) |
| pain | 1.5 | (0.9-2.5) | 0.07 | 1.9 | (1.2-3.2) | 0.007 | 1.2 | 0.4 | (0.6-2.2) |
| **Number of complications** |  |  |  |  |  |  |  |  |  |
| **none** | Reference |  |  | Reference |  |  | Reference |  |  |
| **1** | 3.1 | (1.6-5.9) | 0.00 | 3.4 | (1.7-6.5) | 0.00 | 1.9 | 0.07 | (0.9-4.0) |
| **2** | 3 | (1.5-5.9) | 0.00 | 2.7 | (1.3-5.4) | 0.00 | 1.1 | 0.7 | (0.5-2.6) |
| **≥ 3** | 8.7 | (3.4-22.2) | 0.00 | 8.3 | (3.3-20.9) | 0.00 | 18 | 0.23 | (0.6-4.8) |
| **Risk factors** |  |  |  |  |  |  |  |  |  |
| Hypertension | 1.4 | (0.5-3.4) | 0.44 | 3.5 | (1-12.7) | 0.05 | 2 | 0.3 | (0.4-9.3) |
| Dibetes Mellitus | 1.7 | (1.1-2.8) | 0.01 | 1.2 | (0.7-2.0) | 0.4 | 1.07 | 0.8 | (0.5-1.9) |
| Coronory artery disease | 2.6 | (1.6-4.2) | 0.00 | 1.9 | (1.1-3.1) | 0.01 | 1.1 | 0.7 | (0.6-2.0) |
| Atrial Fibrillation | 2.9 | (1.3-6.2) | 0.00 | 3.3 | (1.4-7.8) | 0.00 | 0.7 | 0.4 | (0.2-1.8) |
| Prior stroke | 2.2 | (1.04-4.9) | 0.03 | 1.2 | (0.5-2.8) | 0.65 | 0.3 | 0.1 | (0.06-1.3) |
| Depression | 1.53 | (0.58-4.08) | 0.38 | 0.9 | (0.3-2.7) | 0.9 | 2.6 | 0.07 | (0.9-7.8) |
| Obesity | 1.15 | (0.7-1.8) | 0.54 | 1.02 | (0.6-1.6) | 0.9 | 0.7 | 0.2 | (0.3-1.2) |
| Dyslipidemia | 0.67 | (0.40-1.11) | 0.12 | 0.9 | (0.5-1.5) | 0.7 | 0.9 | 0.8 | (0.4-1.8) |
| Smoking | 0.48 | (0.24-0.94) | 0.03 | 0.6 | (0.2-1.2) | 0.6 | 0.7 | 0.5 | (0.3-1.8) |
| Smokeless Tobacco | 1.5 | (0.5-4.0) | 0.38 | 0.3 | (0.07-1.63) | 0.17 | 0.3 | 0.3 | (0.04-2.9) |
| Extra cranial carotid stenosis | 0.56 | (0.2-1.3) | 0.21 | 0.4 | (0.15-1.2) | 0.12 | 1.2 | 0.6 | (0.4-3.6) |
| Intra cranial atherosclerotic disease | 0.7 | (0.42-1.15) | 0.16 | 0.8 | (0.4-1.4) | 0.45 | 0.9 | 0.7 | (0.4-1.7) |
| Transient ischemic attack | 0.76 | (0.3-1.9) | 0.57 | 0.3 | (0.08-1.1) | 0.07 | 0.5 | 0.3 | (0.1-2.1) |
| family history of stroke | 0.79 | (0.23-2.6) | 0.71 | 0.6 | (0.2-2.4) | 0.57 | 1.6 | 0.4 | (0.4-6.6) |
| **Recurrent Event** |  |  |  |  |  |  |  |  |  |
| No | Reference |  |  | Reference |  |  | Reference |  |  |
| Yes | 1.02 | (0.5-1.8) | 0.94 | 1.1 | (0.6-2.0) | 0.6 | 1.5 | 0.17 | (0.8-3.0) |
| **Dementia** |  |  |  |  |  |  |  |  |  |
| None | Reference |  |  | Reference |  |  | Reference |  |  |
| Mild | 2.7 | (0.7-9.5) | 0.12 |  |  |  | 3.5 | 0.4-27.7 | (0.00) |
| Moderate and severe | 29.4 | (4.6-61.6) | 0.00 |  |  |  | 16.6 | 2.1-126 | (0.00) |
| **Depression** |  |  |  |  |  |  |  |  |  |
| Non-depressed | Reference |  |  | Reference |  |  | Reference |  |  |
| Depressed | 2.2 | (1.2-3.9) | 0.00 | 5.5 | (2.8-10.7) | 0.00 |  |  |  |

**Annexure 6: Multivariate Logistic regression Analysis of Functional, Cognitive and Psychological Outcomes in Pakistani Stroke Survivors**

| **Variable** | **Adjusted Odds Ratios** | **P-Value** | **95% CI** |
| --- | --- | --- | --- |
| **Functional Outcome** |  |  |  |
| ***Dementia*** |  |  |  |
| None | reference |  |  |
| Mild | 1.9 | 0.30 | 0.5-7.3 |
| Moderate and severe | 19.1 | 0.00 | 5.1-71.8 |
| ***Complication*** |  |  |  |
| None | reference |  |  |
| 1 | 2.2 | 0.04 | 1.02-4.7 |
| 2 | 2.3 | 0.04 | 1.02-5.3 |
| ≥ 3 | 3.6 | 0.02 | 1.2-11.09 |
| ***Age*** |  |  |  |
| ≤ 60 | reference |  |  |
| > 60 | 2.1 | 0.01 | 1.18-4.07 |
| ***Diabetes Mellitus*** |  |  |  |
| No | reference |  |  |
| Yes | 2.1 | 0.02 | 1.08-3.8 |
| **Cognitive Outcome** |  |  |  |
| ***Depression*** |  |  |  |
| No | 1 |  |  |
| Yes | 6.862 | 0.00 | 3.3 - 14.1 |
| ***Complications*** |  |  |  |
| None | 1 |  |  |
| 1 | 3.449 | 0.00 | 1.6-7.1 |
| 2 | 2.803 | 0.01 | 1.2-6.1 |
| ≥ 3 | 4.587 | 0.01 | 1.5-14.0 |
| ***Bed sores*** |  |  |  |
| No | 1 |  |  |
| Yes | 17.137 | 0.01 | 2.0-144.6 |
| ***Atrial Fibrillation*** |  |  |  |
| No | 1 |  |  |
| Yes | 5.121 | 0.00 | 1.9-13.3 |
